# The New Feminism

**Published:** 1920-08-28

**Authors:** 


### THE PUBLIC WELL-BEING.

### The New Feminism.

### DESTRUCTION OF THE FAMILY IDEA.

The discussion which has been proceeding in one of the London newspapers on the subject of marriage is in two particulars distinct from the usual silly-season correspondence, though the subject is peculiar to this familiar period of the year. In the first place, the letter-writers are themselves treating the question with great seriousness, and in numerous instances with marked ability; and, in the second place, Miss Jane Burr, the young American novelist, in her original letter to the Press deliberately raised an issue which has become acute in recent years, especially since the war, and which is none the less serious because its manifestations and its influence have, up to the present, been chiefly "underground." To put it briefly, Miss Burr, who is a feminist of the extreme type, declares roundly that "all women are sick of marriage"; and her mission in life, and for the immediate present in England, appears to be to convert all modern women to her theory of the folly, futility, and wickedness of modern marriage, and the out-of-dateness of family life, which in this age is a grotesque failure and places an intolerable burden on the freedom of her sex. That, as far as can be gathered, fairly and moderately represents her point of view, to which, as she frankly admits, she has in the past personally "converted" an average of three hundred women per annum. The opinion seems to be held by many people that her statements, which are characterised by a certain audacity of expression, are of no importance, and that they should be met with that dangerous boomerang of defence—an attitude of "silent contempt."

In our view, it is very much to be doubted whether this is a wise policy. Miss Burr stands for a kind of feminism which, if it is allowed to penetrate deeply and unresisted into the lives of the people, may before long undermine the whole conception of the family as a basis of civilisation and so strike a deadly blow at the public well-being. Persons who concern themselves in practical questions of social reform and make it their business to keep in close touch with numerous classes of society are well aware that large numbers of girls, particularly those of the middle class, are increasingly attracted by some of the more vicious forms of the no-marriage theory, and the worst of it is that so far this subtle poison has been working its insidious effect not in the open but in secret. It is time the feminists, using the term not in its innocuous sense, were courageously challenged, and the true meaning of their sex advocacy made plain to a world which was never in more need of a sane outlook on the problem of human relationships than at the present time. Most of us are now ready to acknowledge that in the past woman has not had the best of the bargain in the scheme of existence. More than all the fierce campaigns of the militant suffragists, the war forced the general community to realise the fact that women are entitled to a larger responsibility in ordering the affairs of the nation, that they will no longer consent and ought not to consent to play only the part of the domestic drudge, and that their co-operation is urgently called for in all matters that affect the health of the people, the care of children, and the administration of home life. For our own part, also, we have strongly favoured such a reform of the divorce laws as will put women on an equality with men, and no longer leave them at the mercy of cruel or drunken husbands or bound for life to an insane mate. Lord Buckmaster's Matrimonial Causes Bill, which affects the position of women even more than that of men, is itself an admission of the need of reform. It takes a liberal view of marriage and of the hardships and perils involved in marriage; and in so doing it will strengthen instead of weaken the marriage tie. A logical reform of the divorce law is indeed the exact contrary in its purpose, as in its effect, to the schemes of these wild women, who, encouraged by a few "futurist" male thinkers, profess to believe that by destroying the idea of the family they will increase the prestige, the power, the freedom, and the wealth of women. All that they would succeed in doing would be to destroy the security of society and the sanctity of human relationships, and endanger the healthy survival of normal children. The proposal that comes from America for "husbands" and "wives" to live in separate compartments, to meet, say, once a month, and to hand over the care of their children to some apathetic department of bureaucracy is a decadent project which only a decadent nation would accept.

